# The molecular effects of processing on the peanut allergens

**DOI:** 10.1186/2045-7022-4-S2-P10

**Published:** 2014-03-17

**Authors:** Soheila Maleki

**Affiliations:** 1 US Dept of Agriculture, Agricultural Research Service, 1100 Robert E. Lee blvd., New Orleans, LA, USA

## 

The molecular effects of processing on the peanut allergens S. J. Maleki, T. Charles, C.C. Grimm, H. Cheng, B.K. Hurlburt Food Allergy Research, USDA, 1100 Robert E. Lee Blvd., New Orleans, LA 70124, Fax: 504-286-4430, soheila.maleki@ars.usda.gov Food allergy is on the rise and the prevalence of peanut allergy has more than tripled in the U.S. in the last 20 years. Meanwhile, little is known about why certain proteins in foods are allergenic and others are not. Some classic characteristics associated with food allergens are resistance to enzymatic digestion and heat, stimulation of Th2 cell proliferation, IgE binding and, some times, enzymatic function. However, at the molecular level, not much is known about what happens to the allergenicity of food products after processing. We have shown that thermally processed peanut proteins can form higher order structures (oligomers), are less soluble, more resistant to digestive enzymes, and bind higher levels of IgE than raw peanut proteins. We also show that in a majority of patients, roasted peanuts resulted in a higher skin prick test (SPT) reactivity. To determine if processing-induced structural changes in allergens contribute to an increase in IgE binding by roasted peanuts, the major allergens were purified from raw (R),and roasted (Ro) peanuts and the structure and IgE binding to each allergen was compared. While the structure of the allergens purified following roasting did not show significant changes compared to the raw, the IgE binding and SPT to the roasted samples peanuts were higher. Although allergen structure was found to be more important than linear sequence, it is highly likely that the chemical modifications incurred by roasting are more important for enhanced IgE binding and immunogenicity than roasting-induced structural changes of the major peanut allergens. Therefore mass spectroscopic analysis was utilized to identify specific processing-induced chemical modifications of the peanut allergens that may contribute to the observed effects. Understanding the effects of processing at the molecular level and determining the differences in IgE binding to various processed forms of foods may be useful in the development of more specific and improved diagnostic, therapeutic and detection tools and potentially lead to development of processes that can result in reduced allergenicity of a food.

